# Ribavirin Enhances IFN-α Signalling and MxA Expression: A Novel Immune Modulation Mechanism during Treatment of HCV

**DOI:** 10.1371/journal.pone.0027866

**Published:** 2011-11-16

**Authors:** Nigel J. Stevenson, Alison G. Murphy, Nollaig M. Bourke, Catherine A. Keogh, John E. Hegarty, Cliona O'Farrelly

**Affiliations:** 1 School of Biochemistry and Immunology, Trinity College Dublin, Dublin, Ireland; 2 National Liver Transplant Unit, St. Vincent's University Hospital, Dublin, Ireland; Karolinska Institutet, Sweden

## Abstract

The nucleoside analogue Ribavirin significantly increases patient response to IFN-α treatment of HCV, by directly inhibiting viral replication. Recent studies indicate that Ribavirin also regulates immunity and we propose that Ribavirin enhances specific interferon sensitive gene (ISG) expression by amplifying the IFN-α-JAK/STAT pathway. We found that IFN-α-induced STAT1 and STAT3 phosphorylation was increased in hepatocytes co-treated with Ribavirin and IFN-α, compared to IFN-α alone. Ribavirin specifically enhanced IFN-α induced mRNA and protein of the anti-viral mediator MxA, which co-localised with HCV core protein. These novel findings indicate for the first time that Ribavirin, in addition to its viral incorporation, also enhances IFN-α-JAK/STAT signalling, leading to a novel MxA-mediated immuno-modulatory mechanism that may enhance IFN-α anti-viral activity against HCV.

## Introduction

In combination with the anti-viral cytokine interferon (IFN)-α, Ribavirin is used to treat chronic Hepatitis C Virus (HCV) infection. Ribavirin lowers serum alanine aminotransferase levels of HCV infected patients [1], reduces HCV RNA levels [2], [3], decreases patient relapse and more than doubles the sustained virological response (SVR) obtained with IFN-α monotherapy [4]–[6]. Ribavirin (1-β-D-ribofuranosyl-1, 2, 4-triazole-3-carboxamide) is a synthetic guanosine nucleoside analogue, that directly inhibits viral replication by depleting guanosine pools upon inhibition of inosine monophosphate dehydrogenase [7], inhibiting HCV non-structural-5B polymerase [8] and incorporating into the viral genome, leading to error catastrophe [9]. However, in addition to its effect on viral replication, this nucleoside analogue is also thought to have immune modulatory properties, including the regulation of macrophage and T helper (Th) cell produced cytokines, modulation of the Th1/Th2 subset balance and the enhancement of IFN sensitive gene (ISG) expression, suggesting an effect on the JAK/STAT pathway [10]–[15].

Anti-viral responses to IFN-α are mediated by a number of key proteins induced through activation of the JAK/STAT pathway, including double-stranded RNA-activated protein kinase (PKR) and 2′5′-oligoadenylate synthethase (2′5′-OAS), which block translation and degrade viral RNA, respectively. A third critical anti-viral protein is a dynamin-like large guanosine triphosphatase (GTPase), myxovirus resistance gene A (MxA), which is upregulated by IFN-α in many species [16]. Mx proteins were initially discovered in influenza A resistant mice and are thought to mediate innate immunity against numerous RNA viruses, including Hepatitis B [17], [18]. Human Mx proteins (MxA and MxB) are cytoplasmic and involved in vesicle trafficking via their association with the cytoskeleton and endoplasmic reticulum. In fact, this interaction is thought to mediate anti-viral activity by complexing with viral nucleocaspids and trafficking them to cellular locations for isolation or degradation [19]–[21]. This effective anti-viral mechanism is clearly demonstrated in MxA over-expressing mice, which lack functional IFNα/β receptors, but survive normally lethal viral infections despite the inability to mount IFN responses [22].

Since Ribavirin mono-therapy does not clear HCV infection, but enhances patient response to IFN-α, an immuno-modulatory effect is likely, but remains poorly understood. Therefore, the aim of this study was to examine the effect of Ribavirin on IFN-α intracellular signalling, in a bid to further elucidate its mechanism of action. We found that Ribavirin enhanced IFN-α-induced phosphorylation of STAT1 and STAT3 and MxA expression in Huh7 hepatocytes, revealing a novel mechanism of immune regulation by Ribavirin. Furthermore, HCV core protein co-localised with and enhanced MxA protein, implicating MxA as an important anti-viral mediator against HCV. These results are the first to provide a novel insight into the immune modulatory mechanism used by Ribavirin to enhance patient response to IFN-α and may reveal targets within the JAK/STAT pathway for enhancement of current therapy for HCV.

## Methods

### Cell culture

Huh7 cells were grown in 10% FCS, 250 U/ml penicillin, 250 µg/ml streptomycin and 6 µg/ml zeocin DMEM.

### Quantitative (q) RT-PCR

RNA was isolated from cells following the Trizol manufacturer's protocol and cDNA was synthesised using Omniscript (Invitrogen, USA). Each reaction was duplicated, with a total volume of 25 µl∶ 2 µl of cDNA (40 ng/µl), 12.5 µl Sybr green PCR master mix (Roche, Switzerland) and 10.5 µl primer/H_2_O. qRT-PCR was performed using a MX3000P® system (Stratagene Corp, USA), at 95°C for 30 sec, 60°C for 1 min and 72°C for 30 sec. Gene amplifications were normalised to Ribosomal protein 15 (RPS15). Data analysis was carried out using the 2^−ΔΔCT^ method [23]. Livak and Schmittgen, 2001).

Primers:

**MxA-F-**GGTGGTGGTCCCCAGTAATG

**MxA-R-**ACCACGTCCACAACCTTGTCT

**PKR-F**-TCTCAGCAGATACATCAGAGATAAATTCT

**PKR-R**-AGTATACTTTGTTTCTTTCATGTCAGGAA

**2′5′-OAS-F**-AAGAGCCTCATCCGCCTAGTC

**2′5′-OAS-R**-AAATCCCTGGGCTGTGTTGA

**CXCL10-F-**CCAATTTTGTCCACGTGTTGAG

**CXCL10-R-**GCTCCCCTCTGGTTTTAAGGA

**RPS15-F-**CGGACCAAAGCGATCTCTTC

**RPS15-R-**CGCACTGTACAGCTGCATCA

### Transfection

Huh7 cells expressing T7 polymerase were transfected for 12 h using Lipofectamine2000 (Invitrogen, USA), with 5 µg of T7 driven, non-replicating pBRTM/HCV1-3011 DNA construct, containing the entire HCV open reading frame, but lacking the 3′ and 5′ untranslated regions or EV (Invitrogen, USA) in 9 cm plates or with 0.1 µg in poly-D-lysine slides (Becton Dickson, USA) [24].

### Immunoprecipitation and immunoblotting

Huh7 cells were transfected with EV/HCV for 12 h, rested for 2 h in 2% FCS DMEM and then treated with IFN-α (1000 U/ml) and Ribavirin (10 µM). Cells were harvested in lysis buffer (50 mM Hepes, 100 mM NaCl, 1 mM EDTA, 10% glycerol, 0.5% NP40, aprotinin [5 µg/ml], leupeptin [5 µg/ml], PMSF [1 mM] and Na_3_VO_4_ [1 mM]). Protein levels of whole cell lysate were measured using a bicinchoninic acid protein assay (Thermo Scientific) and equal amounts of protein were resolved in SDS-PAGE, followed by transferring to nitrocellulose or polyvinylidene difluoride membranes (Millipore) and blotting with either pSTAT1 (Y701) or pSTAT3 (Y705) (Cell signal), re-blocked and probed for either STAT1 (Cell signal, USA) or STAT3 (Santa Cruz Biotechnologies, USA) and B-actin (Sigma, USA). Blots were stripped of antibody between probing using stripping buffer (Millipore, USA). Lysates were also immunoprecipitated with protein-A/G agarose beads and MxA antibody (Santa Cruz Biotechnologies, USA), before immunoblotting for MxA. Whole cell lysates were probed for HCV core (Abcam, UK) and β-actin (Sigma, USA). Infrared dye-labelled secondary antibodies were used for detection (Li-Cor). Proteins were analysed on an Odyssey Infrared Imaging system using near-infrared fluorescence detection (Li-Cor).

### Immunofluorescence

Huh7 cells were seeded on poly-D-lysine slides for 12 h, before HCV transfection. Cells were fixed for 30 min on ice with 4% paraformaldehyde, washed X3 in PBS and permeabilised using 0.2% Triton x-100 (Sigma, USA) for 15 min at room temperature (RT). Washed X3 in PBS and blocked with PBS containing 4% BSA for 1 h at RT, then incubated with primary antibody in PBS containing 4% BSA overnight at 4°C, followed by incubation with the secondary antibody in 4% BSA for 1 h at RT. Washed X3 with PBS. The slides were mounted using Prolong gold anti-fade reagent containing DAPI (Invitrogen, USA). MxA (Santa Cruz Biotechnologies, USA) was detected using an Alexa 488-conjugated anti-goat antibody (Invitrogen, USA) HCV core (Abcam, USA) was detected using an Alexa 568-conjugated anti-mouse antibody.. Microscopy was performed on an Olympus FV1000 laser scanning confocal microscope, using an UPlanSAPO 60X/1.35 NA oil objective. Cells were digitally sectioned at 0.3 µm per slice and analyzed using the Olympus software.

### Statistical analysis

Data are represented as bar graphs, created from the raw data in Excel or Prism. Groups were then compared using Student's T-test for statistical significance.

## Results

### Ribavirin/IFN-α co-treatment increases STAT activation

**Figure 1:**
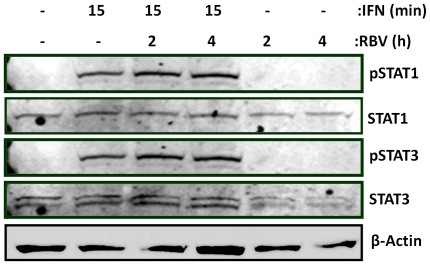
IFN-α induced STAT1 and STAT3 phosphorylation is enhanced in the presence of Ribavirin. Lysates from Huh7 hepatocytes treated with IFN-α for 2 h, Ribavirin for 2 h or both for 2 h were immunoblotted for pSTAT1 and 3 and reprobed for their protein counterparts and β-actin loading control (data representative of three independent experiments).

Since the addition of Ribavirin to IFN-α treatment greatly increases HCV clearance, we wondered if Ribavirin enhances IFN-α-mediated JAK/STAT signal transduction. Initially, we treated Huh7 cells with Ribavirin for 2 and 4 h alone; IFN-α for 15 min alone and Ribavirin pretreatment for 2 and 4 h, before 15 min IFN-α. Lysates were analysed for pSTAT1, STAT1, pSTAT3 and STAT3 protein expression by immunoblotting. As expected, STAT1 and STAT3 phosphorylation occurred after the addition of IFN-α for 15 min. While stimulation with Ribavirin alone did not induce phosphorylation of STAT1 or STAT3, Ribavirin pretreatment amplified subsequent IFN-α-induced STAT1 and STAT3 phosphorylation (Fig. 1). This data demonstrates that Ribavirin enhances IFN-α signal transduction in hepatocytes, which may reveal a mechanism by which Ribavirin increases response to IFN-α therapy in HCV-infected patients.

### Ribavirin specifically enhances induction of MxA mRNA and protein

**Figure 2:**
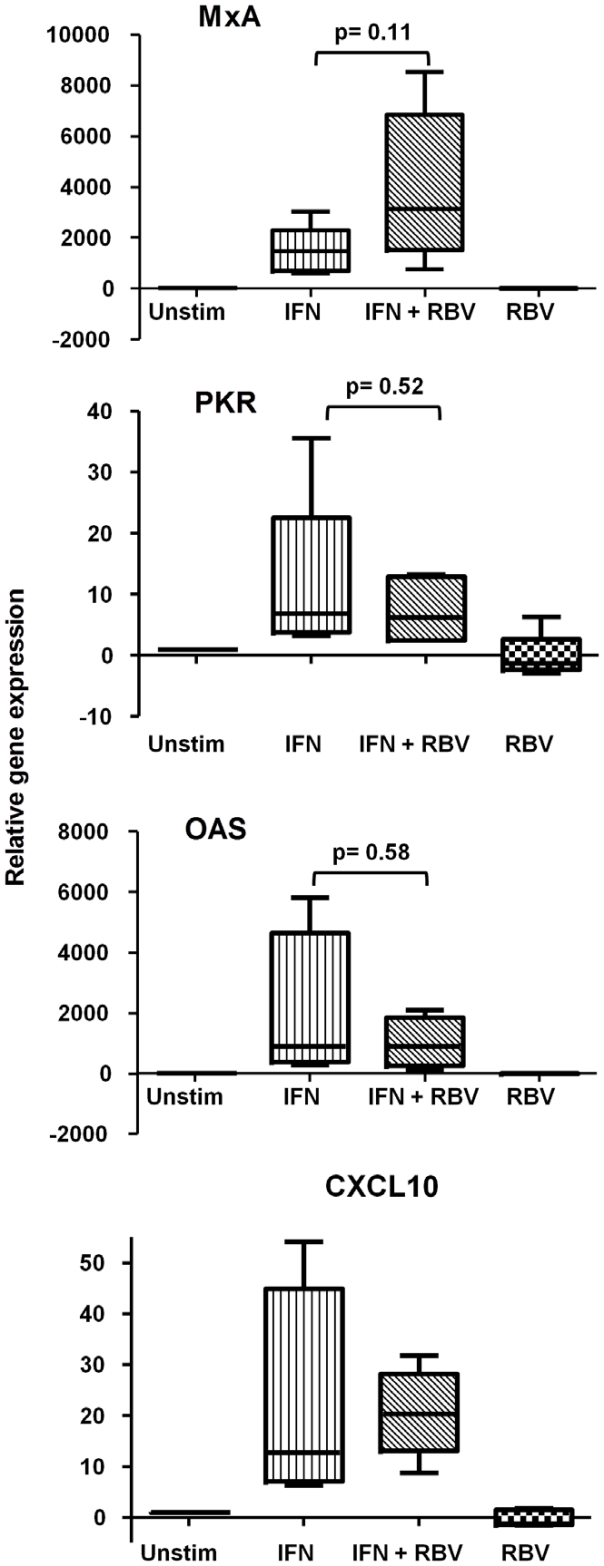
Ribavirin treatment specifically enhances IFN-α-induced MxA mRNA. Quantitative RT-PCR analysis of Huh7 cells treated with IFN-α alone for 2 h, Ribavirin alone for 2 h and RBV and IFN-α together for 2 h. Stimulated values are relative to untreated which are normalized to one (n = 4).

Having observed that Ribavirin increased IFN-α-mediated STAT1 and STAT3 phosphorylation, we subsequently analysed the induction of downstream anti-viral ISGs, including PKR, OAS and MxA. Initially, we treated Huh7 hepatocytes with IFN-α and Ribavirin separately and together for 2 h, before analysing PKR, 2′5′-OAS and MxA mRNA expression by qRT-PCR. To further determine Ribavirin's functional specificity, mRNA levels of the chemokine CXCL10 were measured as a control, since it is strongly upregulated in response to IFN-α, but does not exhibit direct anti-viral activity like PKR, 2′5′-OAS and MxA [25]. IFN-α stimulation strongly induced ISG mRNA expression, while Ribavirin alone had little effect on gene levels. The addition of Ribavirin alongside IFN-α led to a reduction in PKR, 2′5′-OAS and CXCL10 mRNA. However, MxA message was potentiated by Ribavirin/IFN-α co-stimulation (Fig. 2). These results indicate that Ribavirin specifically enhances IFN-α induction of MxA mRNA, via increased STAT phosphorylation, revealing a novel insight into how these therapeutics increase anti-viral responses.

**Figure 3:**
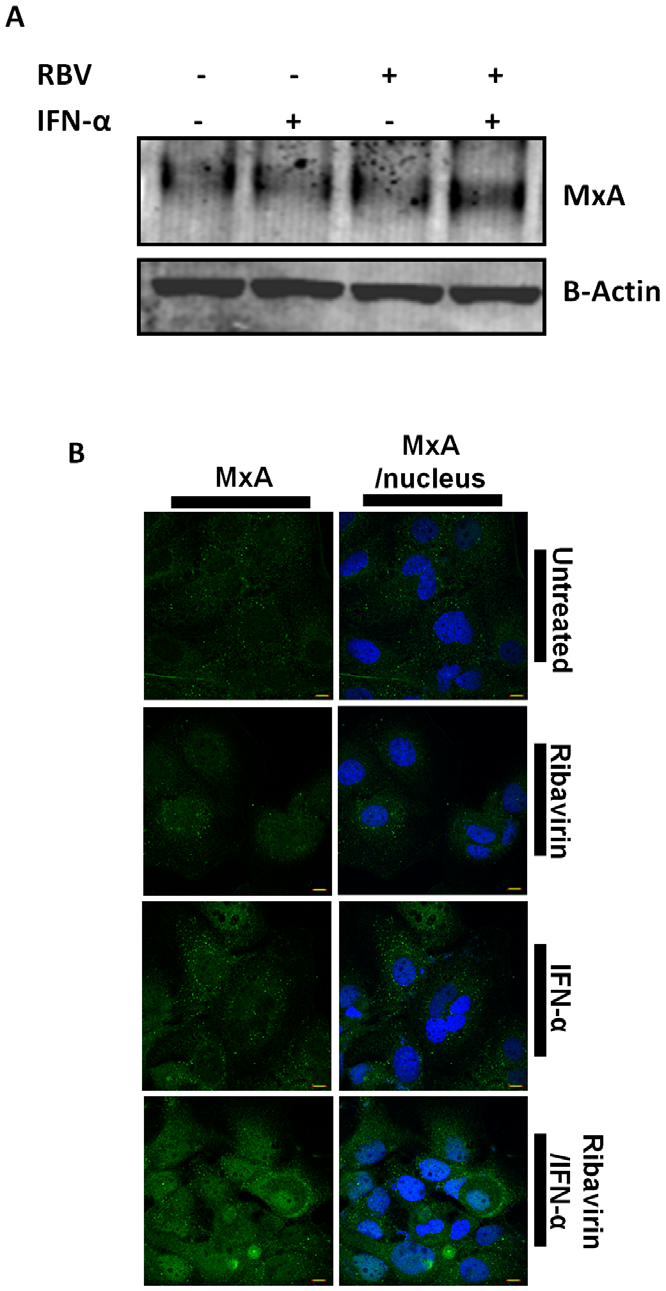
MxA protein expression is enhanced with the addition of IFN-α and Ribavirin. (A) Immunoblot of lysates immunoprecipitated for MxA and probed for MxA from Huh7s treated with IFN-α for 2 h, Ribavirin for 2 h or both for 2 h (n = 3). (B) Confocal micrograph of Huh7s treated with IFN-α for 2 h, Ribavirin for 2 h or both for 2 h (n = 3). Confocal micrograph shows MxA and the nucleus, labelled with Alexa 488 and DAPI, respectively. Bar, 10 µm.

Since we found that a combination of Ribavirin and IFN-α increased MxA mRNA, we wondered whether protein levels were also affected. Therefore, we treated Huh7 cells with IFN-α and Ribavirin separately and together for 2 h, before lysates were harvested, immunoprecipitated for MxA and analysed by Western blotting. While MxA protein was constitutively present in Huh7 s, the addition of IFN-α or Ribavirin alone had no significant impact after 2 h. However, Ribavirin/IFN-α co-treatment mirrored the previously observed mRNA expression by increasing MxA protein levels (Fig. 3A). To further explore the effect of Ribavirin and IFN-α upon MxA protein expression, we treated Huh7 hepatocytes with IFN-α and Ribavirin as before and analysed MxA protein expression by confocal microscopy. We found that Ribavirin and IFN-α together visually increased cytoplasmic MxA protein levels (Fig. 3B), providing support for the MxA protein upregulation observed by immunoblotting (Fig. 3A). These findings indicate that the addition of Ribavirin to IFN-α treatment elevates hepatocyte MxA expression, which may have functional anti-viral consequences during treatment for HCV infection.

### HCV core protein co-localises with MxA

**Figure 4:**
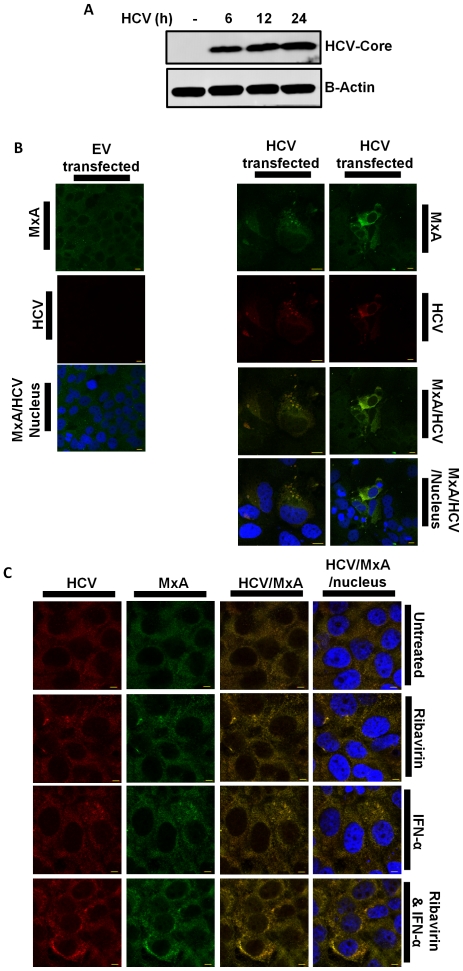
HCV core co-localises with MxA. (A) Immunoblot of lysates from Huh7 cells transfected with HCV-DNA construct for 0, 6, 12 and 24 h, probed with HCV core and β-Actin (n = 3). (B) Confocal micrograph of Huh7s transfected with EV or HCV-DNA construct (n = 3). (C) Huh7s transfected with HCV-DNA construct and treated with IFN-α for 2 h, Ribavirin for 2 h or both for 2 h (n = 4). Confocal micrographs show MxA, HCV core and the nucleus, labelled with Alexa 488, Alexa 568 and DAPI, respectively. Antibody staining is indicated along the side of images Bar, 10 µm (n = 3).

Since these results suggest that MxA is an important anti-viral protein during IFN-α/Ribavirin combination treatment and MxA is thought to bind viral core proteins, targeting them for endosomal isolation, we wondered if MxA also associated with HCV in this manner. Therefore, we initially transfected Huh7 hepatocytes with the non-replicating pBRTM/HCV1-3011 DNA construct over a 24 h time course to determine the optimal transfection time for HCV core protein expression. We found that HCV core protein was strongly expressed within 12 h (Fig. 4A). Next, we transfected Huh7 hepatocytes with a HCV-DNA construct for 12 h and using confocal microscopy, we analysed the association of MxA with HCV. MxA protein levels were increased upon expression of HCV proteins via the transfection of the HCV-DNA construct, indicating a direct MxA-mediated anti-viral response to HCV protein expression. Furthermore, MxA co-localised in a cytoplasmic granular pattern with HCV core (Fig. 4A). We also found that MxA co-localisation with HCV appeared more aggregated, rather than diffuse, upon co-treatment with Ribavirin and IFN-α. (Fig. 4C), suggesting that Ribavirin increases IFN-α-induced MxA expression, thus promoting directional localisation with HCV. These results indicate that MxA targets HCV and suggest that IFN-α/Ribavirin co-treatment may enhance this novel anti-viral process.

## Discussion

Ribavirin increases the response rate of HCV-infected patients to IFN-α therapy, through mechanisms that remain largely unknown, but are thought to reside in its structural similarity to the nucleosides adenosine and guanosine. We found that Ribavirin amplified IFN-α-induced STAT1 and STAT3 phosphorylation and that this combined treatment also increased MxA expression, but had no effect on PKR or 2′5′-OAS, indicating that Ribavirin amplification of JAK/STAT signalling specifically enhances the anti-viral protein MxA. Ribavirin has been shown to upregulate IFN-α receptor expression of hepatocytes [12], perhaps providing a mechanism for our observed increase in IFN-α signal transduction and MxA expression. Interestingly, we observed that in hepatocytes transfected with an HCV-DNA construct, MxA and HCV core protein co-localised in a granular pattern, which may resemble vacuole or endosomal encasement. These findings outline a mechanism by which Ribavirin may enhance anti-viral responses to IFN-α, and suggest that MxA may be fundamental in targeting HCV, thus explaining improved response of patients given Ribavirin in combination with IFN-α.

In agreement with a previous study of chronic HCV infected human livers [26], we found that the presence of HCV increased MxA expression in hepatocytes, suggesting that HCV acts either directly or via paracrine/autocrine induction of IFN-α to induce MxA. Even though MxA has been shown to block the replication of RNA viruses, its exact effect on HCV is undetermined. Frese et al., found that the HCV replicon was not affected by over-expression of MxA and dominant negative MxA did not block IFN-α responses [27]. However, this study did not include Ribavirin; which, as our results suggest, is required for enhanced IFN-α signalling and MxA expression. Furthermore, MxA acts by targeting viruses to endosomal compartments for isolation or degradation, a process that may not lower observed levels of cellular HCV *in vitro* [16]. Indeed, our results show a granular pattern of HCV core:MxA in Huh7 cells, possibly demonstrating this anti-viral mechanism in action. In contrast, Thomas et al., found that IFN-α and Ribavirin inhibit cell cultured HCV infection of Huh7 cells and Itsui et al., found that over-expression of ISGs, including MxA, significantly suppressed the HCV replicon, demonstrating MxA's anti-viral activity against HCV [13], [28].

With the imminent introduction of new protease inhibitors for the treatment of HCV, it was initially thought that IFN-α and Ribavirin might become redundant. However, the phase 2 study for Telaprevir showed that a group of HCV-infected patients given Telaprevir and IFN-α for 12 weeks had SVR of 36%, compared to 60% SVR of those who received Telaprevir, IFN-α plus Ribavirin [29]. These data indicate that Ribavirin will continue to be critical for improved patient response in these novel combination therapies. Together with our results, this suggests that the anti-viral action of protease inhibitors against HCV may further increase the positive effects of Ribavirin upon IFN-α signalling, generating a high level of SVR.

In conclusion, we show that Ribavirin enhances IFN-α JAK/STAT signalling and have identified MxA as a key component of this anti-viral response, which is important in our understanding of how these therapeutics function and in the development of tailored, effective therapeutic care for HCV-infected patients.
